# Supplementary material for: Population genetic structure and predominance of cyclical parthenogenesis in the bird cherry‐oat aphid Rhopalosiphum padi in England
Source: Evol Appl. 2020 Feb 3;13(5):1009–25. doi: 10.1111/eva.12917 (PMC7232763; doi:10.1111/eva.12917)

**Figure S1.** Minion read length frequency histogram.


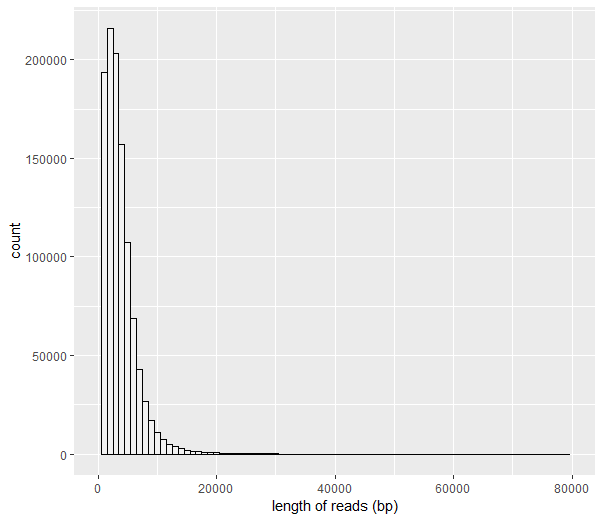


**Figure S2.** BLAST **t**op-hit species distribution.


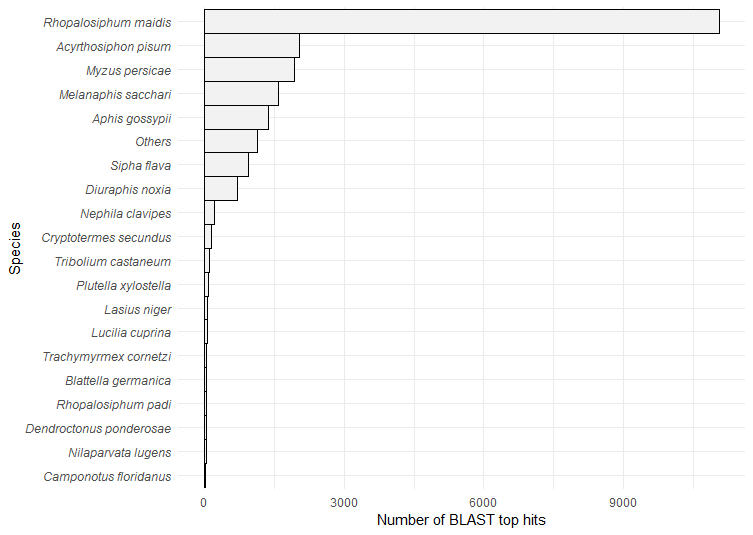


**Figure S3.** Similarity range of the BLAST hits.


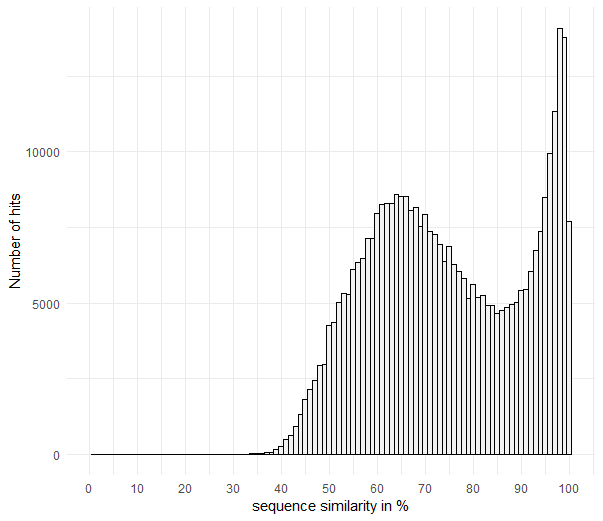


**Figure S4.** Boxplot showing the DNA yield obtained kits from samples collected in different year using different DNA extraction.


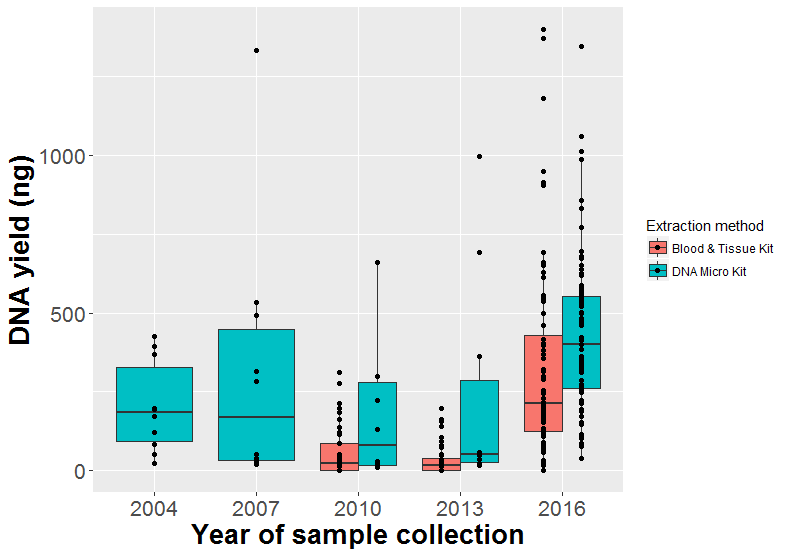


**Figure S5.** Missing data per individual when all samples are analysed together.


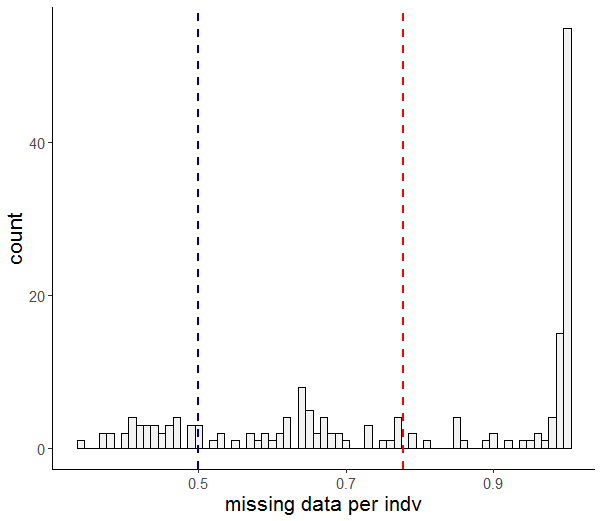


**Figure S6.** Missing data per locus when all samples are analysed together.

**
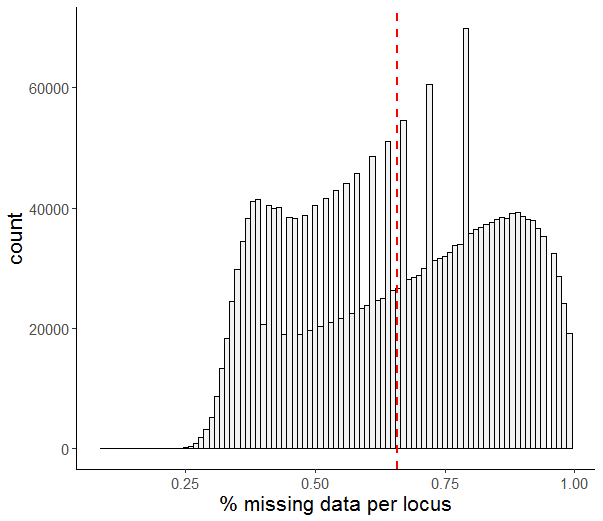
**

**Figure S7.** PCA analysis of all samples data set (FS7) with no evident clustering according to library or genotyping experiment.


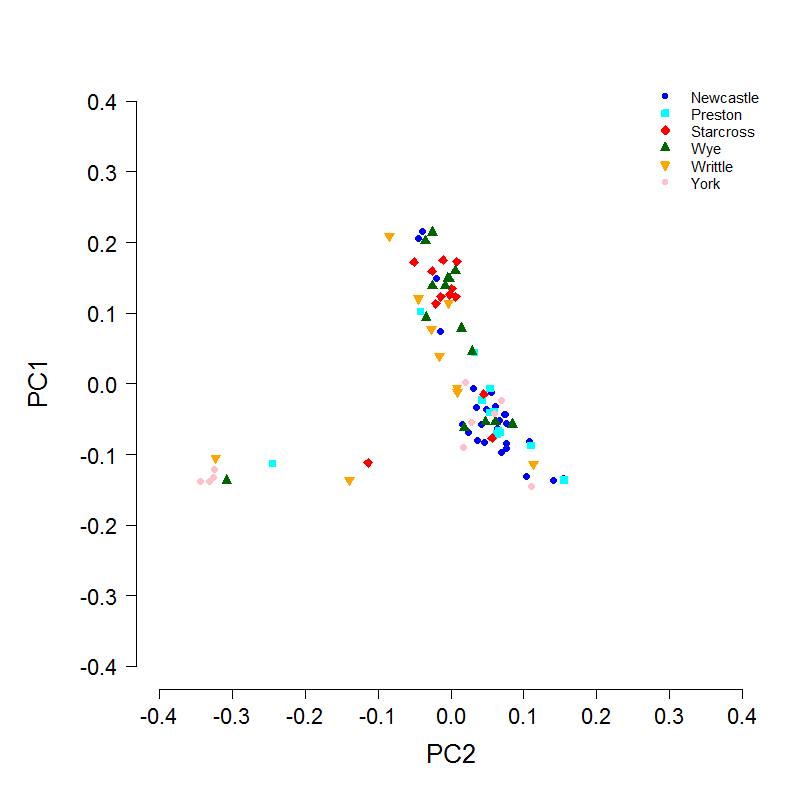


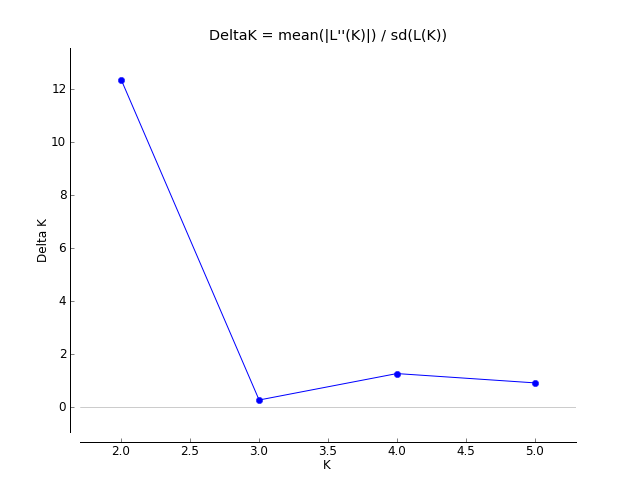
**Figure S8.** Detection of the most likely number of genetic groups (*k*) using Structure and following the statistic Δ(*k*) described in the Evanno *et al*. (2005) method. The likelihood is maximised when *k* = 2.

**Figure S9.** Missing data per locus individual after filtering the Newcastle (A and B) and Starcross (C and D) SNP dataset with the FS2. Dashed vertical lines indicate the mean missing data.

**
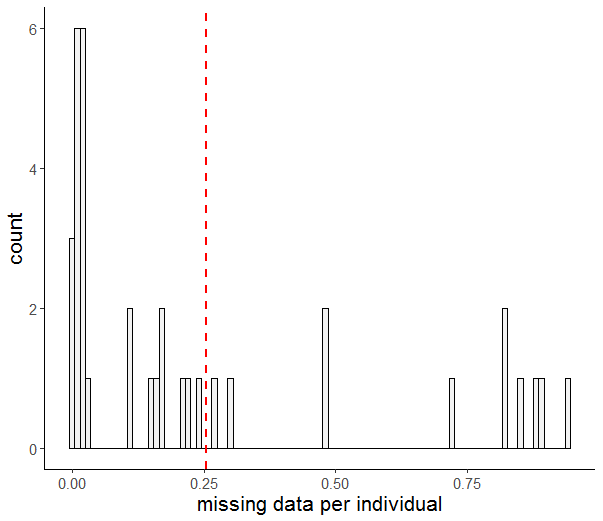

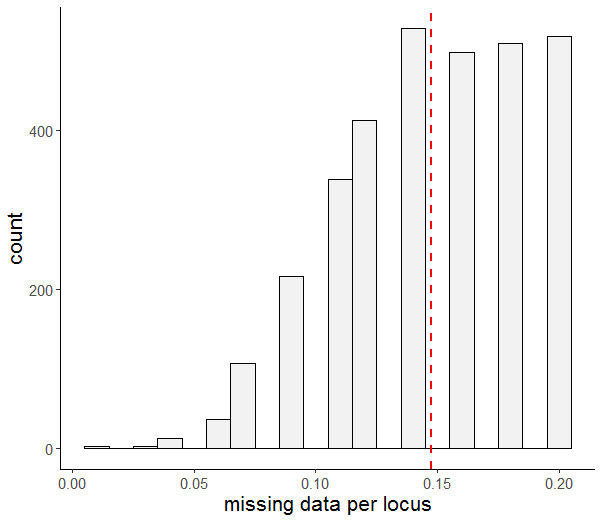
A)** **B)**

**
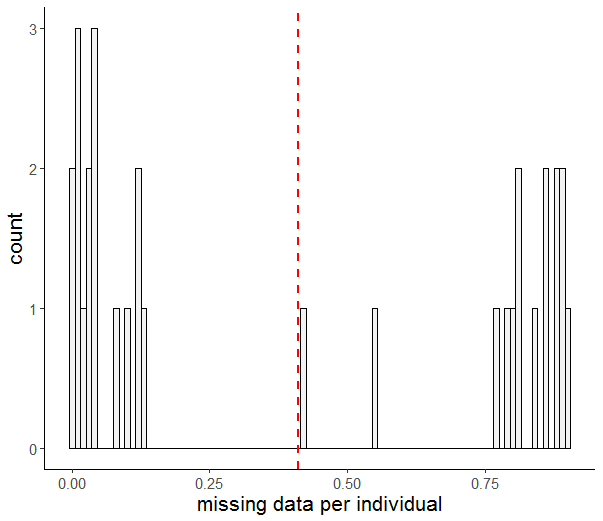
C)** **D)**


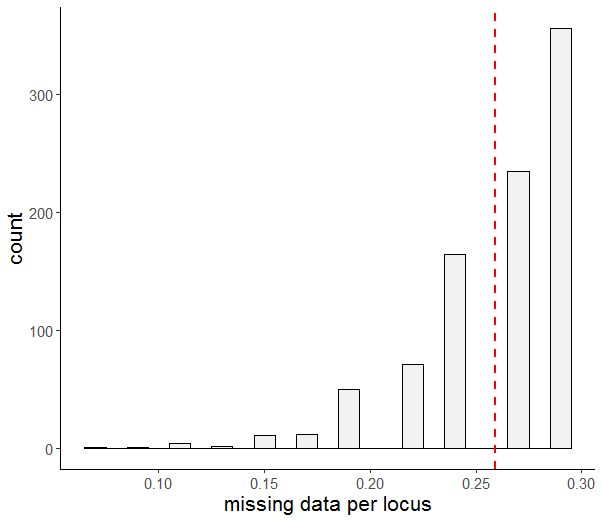

Supplement: Supplementary file 1 [file EVA-13-1009-s001.docx]
