# Supplementary material for: Population genetic structure and predominance of cyclical parthenogenesis in the bird cherry‐oat aphid Rhopalosiphum padi in England
Source: Evol Appl. 2020 Feb 3;13(5):1009–25. doi: 10.1111/eva.12917 (PMC7232763; doi:10.1111/eva.12917)
Supplement: Supplementary file 2 [file EVA-13-1009-s002.docx]

**Table S1.** Samples used in the present study to extract DNA. Date - when the sample was collected in the suction-trap; Extraction method – the kit used to extract DNA from individual aphids; Conc – concentration as measured using Qubit; Total – the amount of DNA yielded by the specimen.

| **Sample** | **Location** | **Trap date** | **Extraction method** | **Conc (ng/ul)** | **Total ammount (ng)** |
| --- | --- | --- | --- | --- | --- |
| RpH1 | Hereford | 2016 | DNA Micro Kit | 6.47 | 194.1 |
| RpH10 | Hereford | 2016 | DNA Micro Kit | 3.76 | 112.8 |
| RpH11 | Hereford | 2016 | DNA Micro Kit | 13.3 | 399 |
| RpH12 | Hereford | 2016 | DNA Micro Kit | 10.4 | 312 |
| RpH13 | Hereford | 2016 | DNA Micro Kit | 3.8 | 114 |
| RpH14 | Hereford | 2016 | DNA Micro Kit | 22.7 | 681 |
| RpH15 | Hereford | 2016 | DNA Micro Kit | 19 | 570 |
| RpH16 | Hereford | 2016 | DNA Micro Kit | 10.5 | 315 |
| RpH17 | Hereford | 2016 | DNA Micro Kit | 11.7 | 351 |
| RpH18 | Hereford | 2016 | DNA Micro Kit | 44.9 | 1347 |
| RpH19 | Hereford | 2016 | DNA Micro Kit | 19.6 | 588 |
| RpH2 | Hereford | 2016 | DNA Micro Kit | 7.16 | 214.8 |
| RpH20 | Hereford | 2016 | DNA Micro Kit | 20.9 | 627 |
| RpH21 | Hereford | 2016 | DNA Micro Kit | 10.7 | 321 |
| RpH22 | Hereford | 2016 | DNA Micro Kit | 17.5 | 525 |
| RpH23 | Hereford | 2016 | DNA Micro Kit | 27.7 | 831 |
| RpH24 | Hereford | 2016 | DNA Micro Kit | 8.63 | 258.9 |
| RpH25 | Hereford | 2016 | DNA Micro Kit | 5.05 | 151.5 |
| RpH3 | Hereford | 2016 | DNA Micro Kit | 10.7 | 321 |
| RpH4 | Hereford | 2016 | DNA Micro Kit | 23.2 | 696 |
| RpH5 | Hereford | 2016 | DNA Micro Kit | 25.7 | 771 |
| RpH6 | Hereford | 2016 | DNA Micro Kit | 8.44 | 253.2 |
| RpH7 | Hereford | 2016 | DNA Micro Kit | 14.1 | 423 |
| RpH8 | Hereford | 2016 | DNA Micro Kit | 11.6 | 348 |
| RpH9 | Hereford | 2016 | DNA Micro Kit | 9.16 | 274.8 |
| RpN36 | Newcastle | 2004 | DNA Micro Kit | 12.3 | 369 |
| RpN37 | Newcastle | 2004 | DNA Micro Kit | 1.75 | 52.5 |
| RpN38 | Newcastle | 2004 | DNA Micro Kit | 14.2 | 426 |
| RpN39 | Newcastle | 2004 | DNA Micro Kit | 13.1 | 393 |
| RpN40 | Newcastle | 2004 | DNA Micro Kit | 6.47 | 194.1 |
| RpN61 | Newcastle | 2004 | DNA Micro Kit | 13.6 | 408 |
| RpN62 | Newcastle | 2004 | DNA Micro Kit | 5.55 | 166.5 |
| RpN63 | Newcastle | 2004 | DNA Micro Kit | 4.6 | 138 |
| RpN64 | Newcastle | 2004 | DNA Micro Kit | 6.66 | 199.8 |
| RpN65 | Newcastle | 2004 | DNA Micro Kit | 3.64 | 109.2 |
| RpN51 | Newcastle | 2007 | DNA Micro Kit | 17.8 | 534 |
| RpN52 | Newcastle | 2007 | DNA Micro Kit | 10.5 | 315 |
| RpN53 | Newcastle | 2007 | DNA Micro Kit | 16.4 | 492 |
| RpN54 | Newcastle | 2007 | DNA Micro Kit | 44.4 | 1332 |
| RpN55 | Newcastle | 2007 | DNA Micro Kit | 9.43 | 282.9 |
| RpN66 | Newcastle | 2007 | DNA Micro Kit | 7.09 | 212.7 |
| RpN67 | Newcastle | 2007 | DNA Micro Kit | 9.65 | 289.5 |
| RpN68 | Newcastle | 2007 | DNA Micro Kit | 23.8 | 714 |
| RpN69 | Newcastle | 2007 | DNA Micro Kit | 19.2 | 576 |
| RpN70 | Newcastle | 2007 | DNA Micro Kit | 17.6 | 528 |
| RpN26 | Newcastle | 2010 | Blood & Tissue Kit | 1.9 | 47.5 |
| RpN27 | Newcastle | 2010 | Blood & Tissue Kit | 7.36 | 184 |
| RpN28 | Newcastle | 2010 | Blood & Tissue Kit | 5.48 | 137 |
| RpN29 | Newcastle | 2010 | Blood & Tissue Kit | 8.56 | 214 |
| RpN30 | Newcastle | 2010 | Blood & Tissue Kit | 4.64 | 116 |
| RpN31 | Newcastle | 2010 | Blood & Tissue Kit | 6.48 | 162 |
| RpN32 | Newcastle | 2010 | Blood & Tissue Kit | 1.3 | 32.5 |
| RpN33 | Newcastle | 2010 | Blood & Tissue Kit | 11.1 | 277.5 |
| RpN34 | Newcastle | 2010 | Blood & Tissue Kit | 12.5 | 312.5 |
| RpN35 | Newcastle | 2010 | Blood & Tissue Kit | 7.88 | 197 |
| RpN46 | Newcastle | 2010 | DNA Micro Kit | 4.36 | 130.8 |
| RpN47 | Newcastle | 2010 | DNA Micro Kit | 9.97 | 299.1 |
| RpN48 | Newcastle | 2010 | DNA Micro Kit | 7.4 | 222 |
| RpN49 | Newcastle | 2010 | DNA Micro Kit | 9.93 | 297.9 |
| RpN50 | Newcastle | 2010 | DNA Micro Kit | 22 | 660 |
| RpN71 | Newcastle | 2010 | DNA Micro Kit | 2.89 | 86.7 |
| RpN72 | Newcastle | 2010 | DNA Micro Kit | 9.54 | 286.2 |
| RpN73 | Newcastle | 2010 | DNA Micro Kit | 7.55 | 226.5 |
| RpN74 | Newcastle | 2010 | DNA Micro Kit | 3.74 | 112.2 |
| RpN75 | Newcastle | 2010 | DNA Micro Kit | 16.2 | 486 |
| RpN16 | Newcastle | 2013 | Blood & Tissue Kit | 0.492 | 12.3 |
| RpN17 | Newcastle | 2013 | Blood & Tissue Kit | 7.92 | 198 |
| RpN18 | Newcastle | 2013 | Blood & Tissue Kit | 0.788 | 19.7 |
| RpN19 | Newcastle | 2013 | Blood & Tissue Kit | 6.28 | 157 |
| RpN20 | Newcastle | 2013 | Blood & Tissue Kit | 3.69 | 92.25 |
| RpN21 | Newcastle | 2013 | Blood & Tissue Kit | 0.732 | 18.3 |
| RpN22 | Newcastle | 2013 | Blood & Tissue Kit | 2 | 50 |
| RpN23 | Newcastle | 2013 | Blood & Tissue Kit | 1.98 | 49.5 |
| RpN24 | Newcastle | 2013 | Blood & Tissue Kit | 6.56 | 164 |
| RpN25 | Newcastle | 2013 | Blood & Tissue Kit | 0.836 | 20.9 |
| RpN56 | Newcastle | 2013 | DNA Micro Kit | 0.696 | 20.88 |
| RpN57 | Newcastle | 2013 | DNA Micro Kit | 1.21 | 36.3 |
| RpN58 | Newcastle | 2013 | DNA Micro Kit | 33.2 | 996 |
| RpN59 | Newcastle | 2013 | DNA Micro Kit | 23.1 | 693 |
| RpN60 | Newcastle | 2013 | DNA Micro Kit | 12.1 | 363 |
| RpN76 | Newcastle | 2013 | DNA Micro Kit | 6.6 | 198 |
| RpN77 | Newcastle | 2013 | DNA Micro Kit | 1.81 | 54.3 |
| RpN78 | Newcastle | 2013 | DNA Micro Kit | 12.2 | 366 |
| RpN79 | Newcastle | 2013 | DNA Micro Kit | 4.61 | 138.3 |
| RpN80 | Newcastle | 2013 | DNA Micro Kit | 2.62 | 78.6 |
| RpN1 | Newcastle | 2016 | Blood & Tissue Kit | 38 | 950 |
| RpN10 | Newcastle | 2016 | Blood & Tissue Kit | 20 | 500 |
| RpN11 | Newcastle | 2016 | Blood & Tissue Kit | 10.2 | 255 |
| RpN12 | Newcastle | 2016 | Blood & Tissue Kit | 9.8 | 245 |
| RpN13 | Newcastle | 2016 | Blood & Tissue Kit | 27.7 | 692.5 |
| RpN14 | Newcastle | 2016 | Blood & Tissue Kit | 19.9 | 497.5 |
| RpN15 | Newcastle | 2016 | Blood & Tissue Kit | 7.44 | 186 |
| RpN2 | Newcastle | 2016 | Blood & Tissue Kit | 47.2 | 1180 |
| RpN3 | Newcastle | 2016 | Blood & Tissue Kit | 36.2 | 905 |
| RpN4 | Newcastle | 2016 | Blood & Tissue Kit | 36.6 | 915 |
| RpN5 | Newcastle | 2016 | Blood & Tissue Kit | 54.8 | 1370 |
| RpN6 | Newcastle | 2016 | Blood & Tissue Kit | 26 | 650 |
| RpN7 | Newcastle | 2016 | Blood & Tissue Kit | 22.2 | 555 |
| RpN8 | Newcastle | 2016 | Blood & Tissue Kit | 26.4 | 660 |
| RpN9 | Newcastle | 2016 | Blood & Tissue Kit | 56 | 1400 |
| RpN41 | Newcastle | 2016 | DNA Micro Kit | 15.8 | 474 |
| RpN42 | Newcastle | 2016 | DNA Micro Kit | 9.16 | 274.8 |
| RpN43 | Newcastle | 2016 | DNA Micro Kit | 32.9 | 987 |
| RpN44 | Newcastle | 2016 | DNA Micro Kit | 8.46 | 253.8 |
| RpN45 | Newcastle | 2016 | DNA Micro Kit | 22.4 | 672 |
| RpP27 | Preston | 2010 | Blood & Tissue Kit | 3.52 | 88 |
| RpP28 | Preston | 2010 | Blood & Tissue Kit | 0.58 | 14.5 |
| RpP29 | Preston | 2010 | Blood & Tissue Kit | 4.88 | 122 |
| RpP30 | Preston | 2010 | Blood & Tissue Kit | <0.01 | 0 |
| RpP31 | Preston | 2010 | Blood & Tissue Kit | <0.01 | 0 |
| RpP32 | Preston | 2010 | Blood & Tissue Kit | 3.41 | 85.25 |
| RpP33 | Preston | 2010 | Blood & Tissue Kit | <0.01 | 0 |
| RpP34 | Preston | 2010 | Blood & Tissue Kit | 1.09 | 27.25 |
| RpP35 | Preston | 2010 | Blood & Tissue Kit | 0.952 | 23.8 |
| RpP36 | Preston | 2010 | Blood & Tissue Kit | 1.45 | 36.25 |
| RpP17 | Preston | 2013 | Blood & Tissue Kit | 0.992 | 24.8 |
| RpP18 | Preston | 2013 | Blood & Tissue Kit | <0.01 | 0 |
| RpP19 | Preston | 2013 | Blood & Tissue Kit | <0.01 | 0 |
| RpP20 | Preston | 2013 | Blood & Tissue Kit | 0.828 | 20.7 |
| RpP21 | Preston | 2013 | Blood & Tissue Kit | 1.49 | 37.25 |
| RpP22 | Preston | 2013 | Blood & Tissue Kit | 3.1 | 77.5 |
| RpP23 | Preston | 2013 | Blood & Tissue Kit | <0.01 | 0 |
| RpP24 | Preston | 2013 | Blood & Tissue Kit | 0.892 | 22.3 |
| RpP25 | Preston | 2013 | Blood & Tissue Kit | 0.512 | 12.8 |
| RpP26 | Preston | 2013 | Blood & Tissue Kit | <0.01 | 0 |
| RpP37 | Preston | 2013 | Blood & Tissue Kit | 1.04 | 26 |
| RpP38 | Preston | 2013 | Blood & Tissue Kit | <0.01 | 0 |
| RpP39 | Preston | 2013 | Blood & Tissue Kit | 4.24 | 106 |
| RpP40 | Preston | 2013 | Blood & Tissue Kit | 3.22 | 80.5 |
| RpP1 | Preston | 2016 | Blood & Tissue Kit | 7.08 | 177 |
| RpP10 | Preston | 2016 | Blood & Tissue Kit | 7.36 | 184 |
| RpP11 | Preston | 2016 | Blood & Tissue Kit | 11.9 | 297.5 |
| RpP12 | Preston | 2016 | Blood & Tissue Kit | 21.5 | 537.5 |
| RpP13 | Preston | 2016 | Blood & Tissue Kit | 8.16 | 204 |
| RpP14 | Preston | 2016 | Blood & Tissue Kit | 11.6 | 290 |
| RpP15 | Preston | 2016 | Blood & Tissue Kit | 12.9 | 322.5 |
| RpP16 | Preston | 2016 | Blood & Tissue Kit | 8.64 | 216 |
| RpP2 | Preston | 2016 | Blood & Tissue Kit | 6.96 | 174 |
| RpP3 | Preston | 2016 | Blood & Tissue Kit | 25.1 | 627.5 |
| RpP4 | Preston | 2016 | Blood & Tissue Kit | 3.43 | 85.75 |
| RpP5 | Preston | 2016 | Blood & Tissue Kit | 24.5 | 612.5 |
| RpP6 | Preston | 2016 | Blood & Tissue Kit | 3.19 | 79.75 |
| RpP7 | Preston | 2016 | Blood & Tissue Kit | 7.04 | 176 |
| RpP8 | Preston | 2016 | Blood & Tissue Kit | 14.8 | 370 |
| RpP9 | Preston | 2016 | Blood & Tissue Kit | 4.76 | 119 |
| RpSX67 | Starcross | 2004 | DNA Micro Kit | 4.09 | 122.7 |
| RpSX68 | Starcross | 2004 | DNA Micro Kit | 0.794 | 23.82 |
| RpSX69 | Starcross | 2004 | DNA Micro Kit | 5.75 | 172.5 |
| RpSX70 | Starcross | 2004 | DNA Micro Kit | 6.56 | 196.8 |
| RpSX71 | Starcross | 2004 | DNA Micro Kit | 2.76 | 82.8 |
| RpSX87 | Starcross | 2004 | DNA Micro Kit | 6.01 | 180.3 |
| RpSX88 | Starcross | 2004 | DNA Micro Kit | 0.58 | 17.4 |
| RpSX89 | Starcross | 2004 | DNA Micro Kit | 1.83 | 54.9 |
| RpSX90 | Starcross | 2004 | DNA Micro Kit | 6.09 | 182.7 |
| RpSX91 | Starcross | 2004 | DNA Micro Kit | 5.33 | 159.9 |
| RpSX77 | Starcross | 2007 | DNA Micro Kit | 1.76 | 52.8 |
| RpSX78 | Starcross | 2007 | DNA Micro Kit | 0.62 | 18.6 |
| RpSX79 | Starcross | 2007 | DNA Micro Kit | 0.973 | 29.19 |
| RpSX80 | Starcross | 2007 | DNA Micro Kit | 0.697 | 20.91 |
| RpSX81 | Starcross | 2007 | DNA Micro Kit | 1.33 | 39.9 |
| RpSX92 | Starcross | 2007 | DNA Micro Kit | 1.27 | 38.1 |
| RpSX93 | Starcross | 2007 | DNA Micro Kit | 1.12 | 33.6 |
| RpSX94 | Starcross | 2007 | DNA Micro Kit | 1.33 | 39.9 |
| RpSX95 | Starcross | 2007 | DNA Micro Kit | 1.25 | 37.5 |
| RpSX96 | Starcross | 2007 | DNA Micro Kit | 1.03 | 30.9 |
| RpSX26 | Starcross | 2010 | Blood & Tissue Kit | 0.46 | 23 |
| RpSX27 | Starcross | 2010 | Blood & Tissue Kit | 0.548 | 27.4 |
| RpSX28 | Starcross | 2010 | Blood & Tissue Kit | <0.01 | 0 |
| RpSX29 | Starcross | 2010 | Blood & Tissue Kit | <0.01 | 0 |
| RpSX30 | Starcross | 2010 | Blood & Tissue Kit | <0.01 | 0 |
| RpSX31 | Starcross | 2010 | Blood & Tissue Kit | 0.852 | 21.3 |
| RpSX32 | Starcross | 2010 | Blood & Tissue Kit | 0.684 | 17.1 |
| RpSX33 | Starcross | 2010 | Blood & Tissue Kit | <0.01 | 0 |
| RpSX34 | Starcross | 2010 | Blood & Tissue Kit | 2.02 | 50.5 |
| RpSX35 | Starcross | 2010 | Blood & Tissue Kit | 0.788 | 19.7 |
| RpSX57 | Starcross | 2010 | Blood & Tissue Kit | <0.01 | 0 |
| RpSX58 | Starcross | 2010 | Blood & Tissue Kit | <0.01 | 0 |
| RpSX59 | Starcross | 2010 | Blood & Tissue Kit | 0.556 | 13.9 |
| RpSX60 | Starcross | 2010 | Blood & Tissue Kit | <0.01 | 0 |
| RpSX61 | Starcross | 2010 | Blood & Tissue Kit | <0.01 | 0 |
| RpSX62 | Starcross | 2010 | Blood & Tissue Kit | 0.716 | 17.9 |
| RpSX63 | Starcross | 2010 | Blood & Tissue Kit | <0.01 | 0 |
| RpSX64 | Starcross | 2010 | Blood & Tissue Kit | 1.86 | 46.5 |
| RpSX65 | Starcross | 2010 | Blood & Tissue Kit | <0.01 | 0 |
| RpSX66 | Starcross | 2010 | Blood & Tissue Kit | <0.01 | 0 |
| RpSX100 | Starcross | 2010 | DNA Micro Kit | 0.516 | 15.48 |
| RpSX101 | Starcross | 2010 | DNA Micro Kit | 0.695 | 20.85 |
| RpSX82 | Starcross | 2010 | DNA Micro Kit | 0.303 | 9.09 |
| RpSX83 | Starcross | 2010 | DNA Micro Kit | 0.446 | 13.38 |
| RpSX84 | Starcross | 2010 | DNA Micro Kit | 0.455 | 13.65 |
| RpSX85 | Starcross | 2010 | DNA Micro Kit | 0.958 | 28.74 |
| RpSX86 | Starcross | 2010 | DNA Micro Kit | 0.721 | 21.63 |
| RpSX97 | Starcross | 2010 | DNA Micro Kit | 0.692 | 20.76 |
| RpSX98 | Starcross | 2010 | DNA Micro Kit | 0.817 | 24.51 |
| RpSX99 | Starcross | 2010 | DNA Micro Kit | 0.674 | 20.22 |
| RpSX16 | Starcross | 2013 | Blood & Tissue Kit | <0.01 | 0 |
| RpSX17 | Starcross | 2013 | Blood & Tissue Kit | 0.984 | 19.68 |
| RpSX18 | Starcross | 2013 | Blood & Tissue Kit | <0.01 | 0 |
| RpSX19 | Starcross | 2013 | Blood & Tissue Kit | <0.01 | 0 |
| RpSX20 | Starcross | 2013 | Blood & Tissue Kit | <0.01 | 0 |
| RpSX21 | Starcross | 2013 | Blood & Tissue Kit | <0.01 | 0 |
| RpSX22 | Starcross | 2013 | Blood & Tissue Kit | <0.01 | 0 |
| RpSX23 | Starcross | 2013 | Blood & Tissue Kit | <0.01 | 0 |
| RpSX24 | Starcross | 2013 | Blood & Tissue Kit | 0.608 | 30.4 |
| RpSX25 | Starcross | 2013 | Blood & Tissue Kit | <0.01 | 0 |
| RpSX47 | Starcross | 2013 | Blood & Tissue Kit | <0.01 | 0 |
| RpSX48 | Starcross | 2013 | Blood & Tissue Kit | <0.01 | 0 |
| RpSX49 | Starcross | 2013 | Blood & Tissue Kit | <0.01 | 0 |
| RpSX50 | Starcross | 2013 | Blood & Tissue Kit | <0.01 | 0 |
| RpSX51 | Starcross | 2013 | Blood & Tissue Kit | <0.01 | 0 |
| RpSX52 | Starcross | 2013 | Blood & Tissue Kit | 5.64 | 141 |
| RpSX53 | Starcross | 2013 | Blood & Tissue Kit | <0.01 | 0 |
| RpSX54 | Starcross | 2013 | Blood & Tissue Kit | 1.45 | 36.25 |
| RpSX55 | Starcross | 2013 | Blood & Tissue Kit | 3 | 75 |
| RpSX56 | Starcross | 2013 | Blood & Tissue Kit | 0.484 | 12.1 |
| RpSX102 | Starcross | 2013 | DNA Micro Kit | 0.535 | 16.05 |
| RpSX103 | Starcross | 2013 | DNA Micro Kit | 0.37 | 11.1 |
| RpSX104 | Starcross | 2013 | DNA Micro Kit | 0.419 | 12.57 |
| RpSX105 | Starcross | 2013 | DNA Micro Kit | 0.624 | 18.72 |
| RpSX106 | Starcross | 2013 | DNA Micro Kit | 0.419 | 12.57 |
| RpSX72 | Starcross | 2013 | DNA Micro Kit | 1.88 | 56.4 |
| RpSX73 | Starcross | 2013 | DNA Micro Kit | 1.66 | 49.8 |
| RpSX74 | Starcross | 2013 | DNA Micro Kit | 0.722 | 21.66 |
| RpSX75 | Starcross | 2013 | DNA Micro Kit | 1.67 | 50.1 |
| RpSX76 | Starcross | 2013 | DNA Micro Kit | 0.595 | 17.85 |
| RpSX1 | Starcross | 2016 | Blood & Tissue Kit | 4.54 | 90.8 |
| RpSX10 | Starcross | 2016 | Blood & Tissue Kit | 6.36 | 127.2 |
| RpSX11 | Starcross | 2016 | Blood & Tissue Kit | 2.95 | 59 |
| RpSX12 | Starcross | 2016 | Blood & Tissue Kit | 5.68 | 113.6 |
| RpSX13 | Starcross | 2016 | Blood & Tissue Kit | 4.03 | 80.6 |
| RpSX14 | Starcross | 2016 | Blood & Tissue Kit | 6.72 | 134.4 |
| RpSX15 | Starcross | 2016 | Blood & Tissue Kit | 5.88 | 117.6 |
| RpSX2 | Starcross | 2016 | Blood & Tissue Kit | 0.828 | 16.56 |
| RpSX3 | Starcross | 2016 | Blood & Tissue Kit | 7.92 | 158.4 |
| RpSX36 | Starcross | 2016 | Blood & Tissue Kit | <0.01 | 0 |
| RpSX37 | Starcross | 2016 | Blood & Tissue Kit | 6.72 | 168 |
| RpSX38 | Starcross | 2016 | Blood & Tissue Kit | 15.2 | 380 |
| RpSX39 | Starcross | 2016 | Blood & Tissue Kit | 6.32 | 158 |
| RpSX4 | Starcross | 2016 | Blood & Tissue Kit | 17.8 | 356 |
| RpSX40 | Starcross | 2016 | Blood & Tissue Kit | 1.08 | 27 |
| RpSX41 | Starcross | 2016 | Blood & Tissue Kit | 5.2 | 130 |
| RpSX42 | Starcross | 2016 | Blood & Tissue Kit | 2.93 | 73.25 |
| RpSX43 | Starcross | 2016 | Blood & Tissue Kit | 1.3 | 32.5 |
| RpSX44 | Starcross | 2016 | Blood & Tissue Kit | 3.22 | 80.5 |
| RpSX45 | Starcross | 2016 | Blood & Tissue Kit | 6.96 | 174 |
| RpSX46 | Starcross | 2016 | Blood & Tissue Kit | 8.08 | 202 |
| RpSX5 | Starcross | 2016 | Blood & Tissue Kit | 11.4 | 228 |
| RpSX6 | Starcross | 2016 | Blood & Tissue Kit | 3.44 | 68.8 |
| RpSX7 | Starcross | 2016 | Blood & Tissue Kit | 5.44 | 108.8 |
| RpSX8 | Starcross | 2016 | Blood & Tissue Kit | 4.4 | 88 |
| RpSX9 | Starcross | 2016 | Blood & Tissue Kit | 1.04 | 20.8 |
| RpWr1 | Writtle | 2016 | DNA Micro Kit | 7.44 | 223.2 |
| RpWr10 | Writtle | 2016 | DNA Micro Kit | 8.72 | 261.6 |
| RpWr11 | Writtle | 2016 | DNA Micro Kit | 12.1 | 363 |
| RpWr12 | Writtle | 2016 | DNA Micro Kit | 25.7 | 771 |
| RpWr13 | Writtle | 2016 | DNA Micro Kit | 18.4 | 552 |
| RpWr14 | Writtle | 2016 | DNA Micro Kit | 20.6 | 618 |
| RpWr15 | Writtle | 2016 | DNA Micro Kit | 28.6 | 858 |
| RpWr16 | Writtle | 2016 | DNA Micro Kit | 17.4 | 522 |
| RpWr17 | Writtle | 2016 | DNA Micro Kit | 11.3 | 339 |
| RpWr18 | Writtle | 2016 | DNA Micro Kit | 4.86 | 145.8 |
| RpWr19 | Writtle | 2016 | DNA Micro Kit | 18.7 | 561 |
| RpWr2 | Writtle | 2016 | DNA Micro Kit | 9.51 | 285.3 |
| RpWr20 | Writtle | 2016 | DNA Micro Kit | 14 | 420 |
| RpWr21 | Writtle | 2016 | DNA Micro Kit | 12.8 | 384 |
| RpWr22 | Writtle | 2016 | DNA Micro Kit | 16.7 | 501 |
| RpWr23 | Writtle | 2016 | DNA Micro Kit | 21.3 | 639 |
| RpWr3 | Writtle | 2016 | DNA Micro Kit | 17.9 | 537 |
| RpWr4 | Writtle | 2016 | DNA Micro Kit | 8.74 | 262.2 |
| RpWr5 | Writtle | 2016 | DNA Micro Kit | 3.43 | 102.9 |
| RpWr6 | Writtle | 2016 | DNA Micro Kit | 5.7 | 171 |
| RpWr7 | Writtle | 2016 | DNA Micro Kit | 8.97 | 269.1 |
| RpWr8 | Writtle | 2016 | DNA Micro Kit | 3.42 | 102.6 |
| RpWr9 | Writtle | 2016 | DNA Micro Kit | 14.1 | 423 |
| RpW1 | Wye | 2016 | Blood & Tissue Kit | 16.1 | 402.5 |
| RpW10 | Wye | 2016 | Blood & Tissue Kit | 18.4 | 460 |
| RpW11 | Wye | 2016 | Blood & Tissue Kit | 18.4 | 460 |
| RpW12 | Wye | 2016 | Blood & Tissue Kit | 16.7 | 417.5 |
| RpW13 | Wye | 2016 | Blood & Tissue Kit | 15.9 | 397.5 |
| RpW14 | Wye | 2016 | Blood & Tissue Kit | 12.6 | 315 |
| RpW15 | Wye | 2016 | Blood & Tissue Kit | 8.68 | 217 |
| RpW2 | Wye | 2016 | Blood & Tissue Kit | 10.2 | 255 |
| RpW3 | Wye | 2016 | Blood & Tissue Kit | 7.68 | 192 |
| RpW4 | Wye | 2016 | Blood & Tissue Kit | 9.92 | 248 |
| RpW5 | Wye | 2016 | Blood & Tissue Kit | 21.8 | 545 |
| RpW6 | Wye | 2016 | Blood & Tissue Kit | 5 | 125 |
| RpW7 | Wye | 2016 | Blood & Tissue Kit | 8.4 | 210 |
| RpW8 | Wye | 2016 | Blood & Tissue Kit | 6.1 | 152.5 |
| RpW9 | Wye | 2016 | Blood & Tissue Kit | 16.1 | 402.5 |
| RpY1 | York | 2016 | DNA Micro Kit | 13.4 | 402 |
| RpY10 | York | 2016 | DNA Micro Kit | 14 | 420 |
| RpY11 | York | 2016 | DNA Micro Kit | 18.2 | 546 |
| RpY12 | York | 2016 | DNA Micro Kit | 15.7 | 471 |
| RpY13 | York | 2016 | DNA Micro Kit | 11.9 | 357 |
| RpY14 | York | 2016 | DNA Micro Kit | 19.3 | 579 |
| RpY15 | York | 2016 | DNA Micro Kit | 11 | 330 |
| RpY16 | York | 2016 | DNA Micro Kit | 19.6 | 588 |
| RpY17 | York | 2016 | DNA Micro Kit | 13.8 | 414 |
| RpY18 | York | 2016 | DNA Micro Kit | 10.6 | 318 |
| RpY19 | York | 2016 | DNA Micro Kit | 11.8 | 354 |
| RpY2 | York | 2016 | DNA Micro Kit | 6.27 | 188.1 |
| RpY20 | York | 2016 | DNA Micro Kit | 8.3 | 249 |
| RpY21 | York | 2016 | DNA Micro Kit | 35.3 | 1059 |
| RpY22 | York | 2016 | DNA Micro Kit | 17.6 | 528 |
| RpY23 | York | 2016 | DNA Micro Kit | 2.53 | 75.9 |
| RpY24 | York | 2016 | DNA Micro Kit | 2.83 | 84.9 |
| RpY25 | York | 2016 | DNA Micro Kit | 12.8 | 384 |
| RpY26 | York | 2016 | DNA Micro Kit | 16.6 | 498 |
| RpY27 | York | 2016 | DNA Micro Kit | 33.8 | 1014 |
| RpY3 | York | 2016 | DNA Micro Kit | 1.27 | 38.1 |
| RpY4 | York | 2016 | DNA Micro Kit | 15.4 | 462 |
| RpY5 | York | 2016 | DNA Micro Kit | 14.1 | 423 |
| RpY6 | York | 2016 | DNA Micro Kit | 21.7 | 651 |
| RpY7 | York | 2016 | DNA Micro Kit | 8.5 | 255 |
| RpY8 | York | 2016 | DNA Micro Kit | 16.6 | 498 |
| RpY9 | York | 2016 | DNA Micro Kit | 16.1 | 483 |

**Table S2.** Details of the samples used for genotype for sequencing (GBS), including the DNA concentration ([DNA]) and amount before and after whole genome amplification (WGA). 2^nd^ WGA refers to the reactions that used the first WGA product as template.

| **Sample** | **Trap date** | **[DNA] (ng/µl)** | **Ammount** | **WGA (ng/µl)** | **Ammount (ng)** | **2^nd^ WGA (ng/µl)** | **Ammount (ng)** | **DNA sequenced (ng)** |
| --- | --- | --- | --- | --- | --- | --- | --- | --- |
| RpN1 | May 2016 | 38 | 950 | 173 | 3,460 |  |  | 800 |
| RpN2 | May 2016 | 47.2 | 1180 | 171 | 3,420 |  |  | 800 |
| RpN3 | May 2016 | 36.2 | 905 | 141 | 2,820 |  |  | 800 |
| RpN4 | May 2016 | 36.6 | 915 | 165 | 3,300 |  |  | 800 |
| RpN5 | June 2016 | 54.8 | 1370 | 197 | 3,940 |  |  | 800 |
| RpN6 | July 2016 | 26 | 650 | 103 | 2,060 |  |  | 800 |
| RpN7 | July 2016 | 22.2 | 555 | 172 | 3,440 |  |  | 800 |
| RpN8 | July 2016 | 26.4 | 660 | 170 | 3,400 |  |  | 800 |
| RpN9 | July 2016 | 56 | 1400 | 152 | 3,040 |  |  | 800 |
| RpN10 | July 2016 | 20 | 500 | 128 | 2,560 |  |  | 800 |
| RpN11 | Oct 2016 | 10.2 | 255 | 136 | 2,720 |  |  | 800 |
| RpN12 | Oct 2016 | 9.8 | 245 | 177 | 3,540 |  |  | 800 |
| RpN13 | Oct 2016 | 27.7 | 692.5 | 156 | 3,120 |  |  | 800 |
| RpN14 | Oct 2016 | 19.9 | 497.5 | 113 | 2,260 |  |  | 800 |
| RpN15 | Oct 2016 | 7.44 | 186 | 102 | 2,040 |  |  | 800 |
| RpN16 | July 2013 | 0.492 | 12.3 | 8.7 | 208.8 | 42 | 840 | 630 |
| RpN17 | July 2013 | 7.92 | 198 | 56.6 | 1358.4 |  |  | 800 |
| RpN18 | July 2013 | 0.788 | 19.7 | 38.4 | 1228.8 |  |  | 768 |
| RpN19 | July 2013 | 6.28 | 157 | 46.4 | 1484.8 |  |  | 696 |
| RpN20 | July 2013 | 3.69 | 92.25 | 41.8 | 1003.2 |  |  | 627 |
| RpN21 | Oct 2013 | 0.732 | 18.3 | 30.6 | 979.2 |  |  | 612 |
| RpN22 | Oct 2013 | 2 | 50 | 32.4 | 1036.8 |  |  | 648 |
| RpN23 | Oct 2013 | 1.98 | 49.5 | 27.8 | 667.2 |  |  | 556 |
| RpN24 | Oct 2013 | 6.56 | 164 | 85 | 2040 |  |  | 800 |
| RpN25 | Oct 2013 | 0.836 | 20.9 | 22.4 | 537.6 |  |  | 537.6 |
| RpN26 | July 2010 | 1.9 | 47.5 | 38.6 | 926.4 |  |  | 772 |
| RpN27 | July 2010 | 7.36 | 184 | 121 | 2904 |  |  | 800 |
| RpN28 | July 2010 | 5.48 | 137 | 110 | 2640 |  |  | 800 |
| RpN29 | July 2010 | 8.56 | 214 | 81 | 1944 |  |  | 800 |
| RpN30 | July 2010 | 4.64 | 116 | 43.2 | 1036.8 |  |  | 648 |
| RpN31 | Oct 2010 | 6.48 | 162 | 109 | 2616 |  |  | 800 |
| RpN32 | Oct 2010 | 1.3 | 32.5 | 17.9 | 572.8 |  |  | 447.5 |
| RpN33 | Oct 2010 | 11.1 | 277.5 | 120 | 2880 |  |  | 800 |
| RpN34 | Oct 2010 | 12.5 | 312.5 | 81.2 | 1948.8 |  |  | 800 |
| RpN35 | Oct 2010 | 7.88 | 197 | 89 | 2136 |  |  | 800 |
| RpN36 | July 2004 | 12.3 | 369 | 87.8 | 2107.2 |  |  | 1930 |
| RpN37 | July 2004 | 1.75 | 52.5 | 34.1 | 750.2 |  |  | 750.2 |
| RpN38 | July 2004 | 14.2 | 426 |  |  |  |  | 400 |
| RpN39 | July 2004 | 13.1 | 393 | 63.2 | 1516.8 |  |  | 1390 |
| RpN40 | July 2004 | 6.47 | 194.1 | 11.5 | 253 | > 60 | 1080 | 1080 |
| RpN50 | July 2010 | 22 | 660 |  |  |  |  | 616 |
| RpN51 | July 2007 | 17.8 | 534 |  |  |  |  | 498.4 |
| RpN52 | July 2007 | 10.5 | 315 | 22.7 | 544.8 |  |  | 500 |
| RpN53 | July 2007 | 16.4 | 492 |  |  |  |  | 460 |
| RpN54 | July 2007 | 44.4 | 1332 |  |  |  |  | 1243 |
| RpN55 | July 2007 | 9.43 | 282.9 | 22.6 | 542.4 |  |  | 497 |
| RpN56 | July 2013 | 0.696 | 20.88 | 7.43 | 178.32 | > 60 | 1080 | 1080 |
| RpN57 | July 2013 | 1.21 | 36.3 | 23.1 | 554.4 |  |  | 508 |
| RpN58 | July 2013 | 33.2 | 996 |  |  |  |  | 930 |
| RpN59 | July 2013 | 23.1 | 693 |  |  |  |  | 647 |
| RpN60 | July 2013 | 12.1 | 363 |  |  |  |  | 339 |
| RpN61 | July 2004 | 13.6 | 408 |  |  |  |  | 381 |
| RpN62 | July 2004 | 5.55 | 166.5 | 11.1 | 244.2 | > 60 | 1080 | 1080 |
| RpN64 | July 2004 | 6.66 | 199.8 | 42.6 | 766.8 |  |  | 937 |
| RpN66 | Aug 2007 | 7.09 | 212.7 | 58.7 | 1408.8 |  |  | 1291 |
| RpN68 | July 2007 | 23.8 | 714 | 23.8 | 714 |  |  | 666 |
| RpN69 | July 2007 | 19.2 | 576 | 19.2 | 576 |  |  | 537.6 |
| RpN70 | Aug 2007 | 17.6 | 528 | 17.6 | 528 |  |  | 493 |
| RpN71 | July 2010 | 2.89 | 86.7 | 38.6 | 926.4 |  |  | 849 |
| RpN72 | July 2010 | 9.54 | 286.2 | > 60 | 1320 |  |  | 1320 |
| RpN73 | July 2010 | 7.55 | 226.5 | > 60 | 1320 |  |  | 1320 |
| RpN74 | July 2010 | 3.74 | 112.2 | 47.1 | 1130.4 |  |  | 1036 |
| RpN75 | July 2010 | 16.2 | 486 |  |  |  |  | 453.6 |
| RpN76 | July 2013 | 6.6 | 198 | 40.5 | 972 |  |  | 891 |
| RpN77 | July 2013 | 1.81 | 54.3 | 10 | 240 | > 60 | 1080 | 1080 |
| RpN78 | July 2013 | 12.2 | 366 |  |  |  |  | 341.6 |
| RpN80 | July 2013 | 2.62 | 78.6 | 18.8 | 451.2 |  |  | 413.6 |
| RpP1 | May 2016 | 7.08 | 177 | 57.6 | 1,152 |  |  | 800 |
| RpP2 | May 2016 | 6.96 | 174 | 40.8 | 816 |  |  | 612 |
| RpP3 | May 2016 | 25.1 | 627.5 | 141 | 2,820 |  |  | 800 |
| RpP4 | May 2016 | 3.43 | 85.75 | 2.32 | 46 | 184 | 3680 | 800 |
| RpP5 | May 2016 | 24.5 | 612.5 | 92.2 | 1,844 |  |  | 800 |
| RpP6 | July 2016 | 3.19 | 79.75 | 4.64 | 93 | 324 | 6480 | 800 |
| RpP7 | July 2016 | 7.04 | 176 | 4.18 | 84 | 244 | 4880 | 800 |
| RpP8 | July 2016 | 14.8 | 370 | 58.4 | 1,168 |  |  | 800 |
| RpP10 | July 2016 | 7.36 | 184 | 63.2 | 1,264 |  |  | 800 |
| RpP11 | Oct 2016 | 11.9 | 297.5 | 157 | 3,140 |  |  | 800 |
| RpP12 | Oct 2016 | 21.5 | 537.5 | 151 | 3,020 |  |  | 800 |
| RpP13 | Oct 2016 | 8.16 | 204 | 79.4 | 1,588 |  |  | 800 |
| RpP14 | Oct 2016 | 11.6 | 290 | 136 | 2,720 |  |  | 800 |
| RpP15 | Oct 2016 | 12.9 | 322.5 | 94.6 | 1,892 |  |  | 800 |
| RpP16 | July 2016 | 8.64 | 216 | 112 | 2,688 |  |  | 800 |
| RpP17 | July 2013 | 0.992 | 24.8 | 46.8 | 1497.6 |  |  | 702 |
| RpP18 | July 2013 | <0.01 | n.a. | 5.54 | 177.28 | 32.4 | 648 | 486 |
| RpP20 | July 2013 | 0.828 | 20.7 | 33.4 | 1068.8 |  |  | 668 |
| RpP21 | July 2013 | 1.49 | 37.25 | 54.8 | 1753.6 |  |  | 657.6 |
| RpP22 | Oct 2013 | 3.1 | 77.5 | 50.6 | 1619.2 |  |  | 759 |
| RpP23 | Oct 2013 | <0.01 | n.a. | 22.2 | 710.4 |  |  | 555 |
| RpP24 | Oct 2013 | 0.892 | 22.3 | 32.4 | 1036.8 |  |  | 648 |
| RpP25 | Oct 2013 | 0.512 | 12.8 | 21.6 | 691.2 |  |  | 540 |
| RpP27 | July 2010 | 3.52 | 88 | 53.4 | 1708.8 |  |  | 800 |
| RpP28 | July 2010 | 0.58 | 14.5 | 19.6 | 627.2 |  |  | 490 |
| RpP29 | July 2010 | 4.88 | 122 | 54.8 | 1753.6 |  |  | 800 |
| RpP30 | July 2010 | <0.01 | n.a. | 17.6 | 563.2 |  |  | 563.2 |
| RpP31 | July 2010 | <0.01 | n.a. | 19.8 | 633.6 |  |  | 495 |
| RpP32 | Oct 2010 | 3.41 | 85.25 | 41.8 | 1337.6 |  |  | 627 |
| RpP33 | Oct 2010 | <0.01 | n.a. | 14.6 | 467.2 | 31.8 | 636 | 636 |
| RpP34 | Oct 2010 | 1.09 | 27.25 | 40.8 | 1305.6 |  |  | 612 |
| RpP35 | Oct 2010 | 0.952 | 23.8 | 19.6 | 627.2 |  |  | 490 |
| RpP36 | Oct 2010 | 1.45 | 36.25 | 22.6 | 723.2 |  |  | 565 |
| RpP37 | July 2013 | 1.04 | 26 | 7.48 | 239.36 | 35.8 | 859.2 | 537 |
| RpP40 | Oct 2013 | 3.22 | 80.5 | 41.4 | 1324.8 |  |  | 621 |
| RpSX1 | Apr 2016 | 4.54 | 90.8 | 123 | 2,460 |  |  | 800 |
| RpSX3 | May 2016 | 7.92 | 158.4 | 143 | 2,860 |  |  | 800 |
| RpSX9 | July 2016 | 1.04 | 20.8 | 5.98 | 120 | 312 | 6240 | 800 |
| RpSX11 | Oct 2016 | 2.95 | 59 | 39.6 | 792 | 498 | 9960 | 800 |
| RpSX13 | Oct 2016 | 4.03 | 80.6 | 46.8 | 936 | 470 | 9400 | 800 |
| RpSX16 | July 2013 | <0.01 | n.a. | 10.8 | 346 |  |  | 345.6 |
| RpSX17 | July 2013 | 0.984 | 19.68 | 34.2 | 1,094 |  |  | 684 |
| RpSX24 | Oct 2013 | 0.608 | 30.4 | 27 | 864 |  |  | 675 |
| RpSX27 | July 2010 | 0.548 | 27.4 | 39.8 | 1,274 |  |  | 796 |
| RpSX31 | Oct 2010 | 0.852 | 21.3 | 64 | 2,048 |  |  | 800 |
| RpSX32 | Oct 2010 | 0.684 | 17.1 | 47.4 | 1,517 |  |  | 711 |
| RpSX34 | Oct 2010 | 2.02 | 50.5 | 40 | 1,280 |  |  | 800 |
| RpSX35 | Oct 2010 | 0.788 | 19.7 | 23.6 | 755 |  |  | 590 |
| RpSX37 | May 2016 | 6.72 | 168 | 124 | 2,976 |  |  | 800 |
| RpSX38 | May 2016 | 15.2 | 380 | 135 | 3,240 |  |  | 800 |
| RpSX39 | May 2016 | 6.32 | 158 | 125 | 3,000 |  |  | 800 |
| RpSX40 | July 2016 | 1.08 | 27 | 82 | 1,968 |  |  | 800 |
| RpSX41 | July 2016 | 5.2 | 130 | 117 | 2,808 |  |  | 800 |
| RpSX42 | July 2016 | 2.93 | 73.25 | 92.6 | 2,222 |  |  | 800 |
| RpSX43 | July 2016 | 1.3 | 32.5 | 73.2 | 1,757 |  |  | 800 |
| RpSX44 | Oct 2016 | 3.22 | 80.5 | 90 | 2,160 |  |  | 800 |
| RpSX45 | Oct 2016 | 6.96 | 174 | 126 | 3,024 |  |  | 800 |
| RpSX46 | Oct 2016 | 8.08 | 202 | 117 | 2,808 |  |  | 800 |
| RpSX49 | July 2013 | <0.01 | n.a. | 4.4 | 140.8 | 338 | 6760 | 800 |
| RpSX50 | July 2013 | <0.01 | n.a. | 9.06 | 289.92 | 140 | 2800 | 800 |
| RpSX51 | July 2013 | <0.01 | n.a. | 10.7 | 342.4 | 414 | 8280 | 800 |
| RpSX52 | Oct 2013 | 5.64 | 141 | 55.4 | 1772.8 |  |  | 831 |
| RpSX54 | Oct 2013 | 1.45 | 36.25 | 49.8 | 1593.6 |  |  | 747 |
| RpSX55 | Oct 2013 | 3 | 75 | 56.6 | 1811.2 |  |  | 800 |
| RpSX56 | Oct 2013 | 0.484 | 12.1 | 26 | 832 |  |  | 520 |
| RpSX58 | July 2010 | <0.01 | n.a. | 12.4 | 396.8 | 222 | 4440 | 800 |
| RpSX59 | July 2010 | 0.556 | 13.9 | 2.36 | 75.52 | 45 | 900 | 675 |
| RpSX60 | July 2010 | <0.01 | n.a. | 15.8 | 505.6 | 88.6 | 1772 | 800 |
| RpSX64 | Oct 2010 | 1.86 | 46.5 | 27.2 | 870.4 |  |  | 680 |
| RpSX66 | July 2010 | <0.01 | n.a. | 3.9 | 124.8 | 54.2 | 1300.8 | 813 |
| RpSX68 | July 2004 | 0.794 | 23.82 | >60 | 1320 |  |  | 1320 |
| RpSX69 | July 2004 | 5.75 | 172.5 | >60 | 1320 |  |  | 1320 |
| RpSX70 | July 2004 | 6.56 | 196.8 | 50 | 1200 |  |  | 1100 |
| RpSX71 | July 2004 | 2.76 | 82.8 | 34.8 | 835.2 |  |  | 765.6 |
| RpSX72 | July 2013 | 1.88 | 56.4 | 33.2 | 796.8 |  |  | 1144 |
| RpSX73 | July 2013 | 1.66 | 49.8 | 24.1 | 578.4 |  |  | 530.2 |
| RpSX74 | July 2013 | 0.722 | 21.66 | >60 | 1320 |  |  | 1276 |
| RpSX75 | July 2013 | 1.67 | 50.1 | 45.7 | 1096.8 |  |  | 532.4 |
| RpSX76 | July 2013 | 0.595 | 17.85 | 44.1 | 1058.4 |  |  | 1020 |
| RpSX77 | July 2007 | 1.76 | 52.8 | 75.4 | 1809.6 |  |  | 1658.8 |
| RpSX78 | July 2007 | 0.62 | 18.6 | 53.2 | 1276.8 |  |  | 1170.4 |
| RpSX79 | July 2007 | 0.973 | 29.19 | 57 | 1368 |  |  | 1254 |
| RpSX80 | July 2007 | 0.697 | 20.91 | 55 | 1320 |  |  | 1210 |
| RpSX81 | July 2007 | 1.33 | 39.9 | >60 | 1320 |  |  | 1320 |
| RpSX82 | July 2010 | 0.303 | 9.09 | 34.3 | 823.2 |  |  | 754.6 |
| RpSX83 | July 2010 | 0.446 | 13.38 | 59 | 1416 |  |  | 1298 |
| RpSX84 | July 2010 | 0.455 | 13.65 | >60 | 1320 |  |  | 1320 |
| RpSX85 | July 2010 | 0.958 | 28.74 | 48.7 | 1168.8 |  |  | 1071.4 |
| RpSX86 | July 2010 | 0.721 | 21.63 | 43.2 | 1036.8 |  |  | 950.4 |
| RpSX87 | July 2004 | 6.01 | 180.3 | 49.7 | 1192.8 |  |  | 1093.4 |
| RpSX88 | July 2004 | 0.58 | 17.4 | 26.9 | 646 |  |  | 591.8 |
| RpSX89 | July 2004 | 1.83 | 54.9 | >60 | 1320 |  |  | 1320 |
| RpSX90 | July 2004 | 6.09 | 182.7 | 50 | 1200 |  |  | 1100 |
| RpSX91 | July 2004 | 5.33 | 159.9 | >60 | 1320 |  |  | 1320 |
| RpSX92 | July 2007 | 1.27 | 38.1 | 26.7 | 640.8 |  |  | 587.4 |
| RpSX93 | July 2007 | 1.12 | 33.6 | 25.4 | 609.6 |  |  | 558.8 |
| RpSX94 | July 2007 | 1.33 | 39.9 | >60 | 1320 |  |  | 1320 |
| RpSX95 | July 2007 | 1.25 | 37.5 | 55 | 1320 |  |  | 1210 |
| RpSX96 | July 2007 | 1.03 | 30.9 | 32.3 | 775.2 |  |  | 710.6 |
| RpSX97 | July 2010 | 0.692 | 20.76 | 31.8 | 763.2 |  |  | 699.6 |
| RpSX98 | July 2010 | 0.817 | 24.51 | 58 | 1392 |  |  | 1276 |
| RpSX99 | July 2010 | 0.674 | 20.22 | 39 | 936 |  |  | 858 |
| RpSX100 | July 2010 | 0.516 | 15.48 | 19.8 | 475.2 |  |  | 435.6 |
| RpSX101 | July 2010 | 0.695 | 20.85 | 15.3 | 367.2 |  |  | 336.6 |
| RpSX103 | July 2013 | 0.37 | 11.1 | >60 | 1320 |  |  | 1320 |
| RpSX104 | July 2013 | 0.419 | 12.57 | 22 | 528 |  |  | 488.4 |
| RpSX105 | July 2013 | 0.624 | 18.72 | 13.9 | 333.6 |  |  | 320 |
| RpSX106 | July 2013 | 0.419 | 12.57 | >60 | 1320 |  |  | 794.2 |
| RpW1 | Apr 2016 | 16.1 | 402.5 | 220 | 4,400 |  |  | 800 |
| RpW2 | May 2016 | 10.2 | 255 | 131 | 2,620 |  |  | 800 |
| RpW3 | May 2016 | 7.68 | 192 | 82.6 | 1,652 |  |  | 800 |
| RpW4 | May 2016 | 9.92 | 248 | 168 | 3,360 |  |  | 800 |
| RpW5 | May 2016 | 21.8 | 545 | 212 | 4,240 |  |  | 800 |
| RpW6 | July 2016 | 5 | 125 | 119 | 2,380 |  |  | 800 |
| RpW7 | July 2016 | 8.4 | 210 | 177 | 3,540 |  |  | 800 |
| RpW8 | July 2016 | 6.1 | 152.5 | 97.2 | 1,944 |  |  | 800 |
| RpW9 | July 2016 | 16.1 | 402.5 | 208 | 4,160 |  |  | 800 |
| RpW10 | July 2016 | 18.4 | 460 | 206 | 4,120 |  |  | 800 |
| RpW11 | Oct 2016 | 18.4 | 460 | 248 | 4,960 |  |  | 800 |
| RpW12 | Oct 2016 | 16.7 | 417.5 | 258 | 5,160 |  |  | 800 |
| RpW13 | Oct 2016 | 15.9 | 397.5 | 230 | 4,600 |  |  | 800 |
| RpW14 | Oct 2016 | 12.6 | 315 | 206 | 4,120 |  |  | 800 |
| RpW15 | Oct 2016 | 8.68 | 217 | 188 | 3,760 |  |  | 800 |
| RpWr3 | July 2016 | 17.9 | 537 |  |  |  |  | 501.2 |
| RpWr9 | July 2016 | 14.1 | 423 |  |  |  |  | 395 |
| RpWr12 | July 2016 | 25.7 | 771 |  |  |  |  | 719.6 |
| RpWr13 | July 2016 | 18.4 | 552 |  |  |  |  | 515.2 |
| RpWr14 | July 2016 | 20.6 | 618 |  |  |  |  | 576.8 |
| RpWr15 | July 2016 | 28.6 | 858 |  |  |  |  | 800.8 |
| RpWr16 | Aug 2016 | 17.4 | 522 |  |  |  |  | 487.2 |
| RpWr19 | Aug 2016 | 18.7 | 561 |  |  |  |  | 523.6 |
| RpWr22 | July 2016 | 16.7 | 501 |  |  |  |  | 467.6 |
| RpWr23 | July 2016 | 21.3 | 639 |  |  |  |  | 596.4 |
| RpY6 | July 2016 | 21.7 | 651 |  |  |  |  | 607.6 |
| RpY8 | July 2016 | 16.6 | 498 |  |  |  |  | 464.8 |
| RpY9 | July 2016 | 16.1 | 483 |  |  |  |  | 450.8 |
| RpY11 | July 2016 | 18.2 | 546 |  |  |  |  | 509.6 |
| RpY14 | Aug 2016 | 19.3 | 579 |  |  |  |  | 540.4 |
| RpY16 | Aug 2016 | 19.6 | 588 |  |  |  |  | 548.8 |
| RpY21 | Aug 2016 | 35.3 | 1059 |  |  |  |  | 988.4 |
| RpY22 | Aug 2016 | 17.6 | 528 |  |  |  |  | 492.8 |
| RpY26 | Aug 2016 | 16.6 | 498 |  |  |  |  | 464.8 |
| RpY27 | Aug 2016 | 33.8 | 1014 |  |  |  |  | 946.4 |

**Table S3.** Filtering schemes for the different data sets used in the population structure analyses: A) all samples included; B) samples from Newcastle only; C) samples from Starcross only. The order of rows indicates the sequential filters applied to the data. minDP – include only genotypes with depth greater or equal to the value; minQ – include sites with quality above the value; mac – include sites with minor allele count greater or equal to the value; geno – retain sites that have been successfully genotyped in the given proportion of individuals (max-missing filtering option in vcftools); imiss – retain individuals with a proportion of missing data smaller than the value. FS highlighted in green show the datasets used in the analyses.

**A)**

| Filter | fs1 | fs2 | FS3 | FS4 | FS5 | FS6 | FS7 |
| --- | --- | --- | --- | --- | --- | --- | --- |
| Missing data |  |  |  |  |  | geno > 50% | geno > 50%  remove-indels |
| Low-confidence SNP call |  |  | minDP > 5  minQ > 20  mac > 3 | minDP > 5  minQ > 20  mac > 3 | minDP > 5  minQ > 20  mac > 3 | mac > 3  minQ > 30  minDP > 3 | mac >3  minQ > 20  minDP > 3 |
| Missing data | geno > 90%  remove-indels  imiss < 25% / < 50%  Not done imiss for all samples as it is not worthy | imiss < 78%  geno > 90% | geno > 90% | imiss < 90%  geno > 90% | geno > 15%  imiss < 95%  geno > 60%  imiss < 80%  geno > 70%  imiss < 75%  geno > 80% | imiss < 95%  geno > 75% | imiss < 60%  geno > 90% |
| Low-confidence SNP call |  | minDP > 5  minQ > 20  mac > 3 |  |  |  |  | minDP > 5 |
| Missing data |  |  |  |  |  |  | geno > 95% |
| INFO filters | Remove-indels | Remove-indels |  |  |  | remove-indels | thin 2000 |
| SNPs | 2 | 63,187 (2,287,871) | 0 | 1704 | 3244 (2,287,871) | 1526 | 4802 |
| Individuals | Not done | 84 (175) | 175 | 60 (175) | 67 (175) | 91 | 86 |

**B)**

| Filter | fs1 | fs2 | FS3 | FS4 |
| --- | --- | --- | --- | --- |
| Missing data | geno > 50%  remove-indels | remove-indels  geno > 50% | geno > 50%  imiss < 99%  geno > 75% |  |
| Low-confidence SNP call |  | min-meanDP > 5  mac > 3  minQ > 20 | minDP > 3  mac > 3  minQ > 20 | minDP > 3  mac > 3  minQ > 20 |
| Missing data | imiss < 75%  geno > 90%  thin 2Kb | imiss < 95%  geno > 80%  thin 2kb | geno > 50%  thin 2kb  remove-indels  imiss < 90% | thin 2kb  geno > 30%  imiss < 99%  remove-indels  imiss < 95  geno > 60%  geno > 70% |
| Low-confidence SNP call | min-meanDP > 5  mac > 3  minQ > 20 |  |  |  |
| Missing data |  |  |  |  |
| INFO filters |  |  |  |  |
| SNPs | 1746 (< 10% missing per locus) | 3186 (< 20% missing per locus) | 5277 (< 50% missing per locus) | 359 (< 30% missing per locus) |
| Individuals | 28 (< 50% missing per indv) | 36 (0-90% missing per indv) | 29 (0-85% missing per indv) | 33 (0 – 91% missing per indv) |

**C)**

| Filter | FS1 | FS2 | FS3 | FS4 |
| --- | --- | --- | --- | --- |
| Missing data | geno > 50%  remove-indels | remove-indels  geno > 25% | geno > 25%  imiss < 99%  geno > 50% |  |
| Low-confidence SNP call |  | min-meanDP > 5  mac > 3  minQ > 20 | minDP > 3  mac > 3  minQ > 20 | minDP > 3  mac > 3  minQ > 20 |
| Missing data | imiss < 95%  thin 2kb | imiss < 95%  geno > 70%  think 2K | geno > 60%  thin 2kb  remove-indels  imiss < 90% | thin 2kb  geno > 20%  imiss < 99%  remove-indels  geno > 60%  imiss < 95% |
| Low-confidence SNP call | minDP > 3  mac > 3  minQ > 20 |  |  |  |
| Missing data |  |  |  |  |
| INFO filters |  |  |  |  |
| SNPs | 89 (< 90% missing per locus) | 907 (< 30% missing per locus) | 1638 (< 40% missing data per locus) | 359 (< 50% missing per locus) |
| Individuals | 46 (0-99% missing per indv) | 31 (0-89% missing data per indv) | 18 (< 75% missing data per indv) | 27 (0-92% missing per indv) |

**Table S4.** Sequencing results for the aphids used in GBS showing the number of reads that passed the QC, the number and percentage of reads aligned to the *R. padi* genome.

| **Sample** | **Trap date** | **N reads** | **Mapped reads** | **Percentage mapped** |
| --- | --- | --- | --- | --- |
| RpN1 | May 2016 | 4995472 | 4954101 | 99.17% |
| RpN10 | July 2016 | 5200744 | 4826984 | 92.81% |
| RpN11 | October 2016 | 5167156 | 5130989 | 99.30% |
| RpN12 | October 2016 | 5714318 | 5675868 | 99.33% |
| RpN13 | October 2016 | 8999479 | 8936557 | 99.30% |
| RpN14 | October 2016 | 8738807 | 8672698 | 99.24% |
| RpN15 | October 2016 | 9016847 | 8580189 | 95.16% |
| RpN16 | July 2013 | 1961831 | 87707 | 4.47% |
| RpN2 | May 2016 | 4819781 | 4786969 | 99.32% |
| RpN23 | October 2013 | 339022 | 252469 | 74.47% |
| RpN26 | July 2010 | 276 | 0 | 0.00% |
| RpN27 | July 2010 | 6925877 | 6713675 | 96.94% |
| RpN28 | July 2010 | 8563521 | 8464873 | 98.85% |
| RpN29 | July 2010 | 6146045 | 6075966 | 98.86% |
| RpN3 | May 2016 | 5244950 | 5066806 | 96.60% |
| RpN30 | July 2010 | 2835505 | 2449152 | 86.37% |
| RpN31 | October 2010 | 10039372 | 9896932 | 98.58% |
| RpN33 | October 2010 | 9595798 | 9258270 | 96.48% |
| RpN34 | October 2010 | 9714165 | 9614719 | 98.98% |
| RpN36 | July 2004 | 5287189 | 5118021 | 96.80% |
| RpN37 | July 2004 | 1611708 | 188437 | 11.69% |
| RpN38 | July 2004 | 3192066 | 3154267 | 98.82% |
| RpN39 | July 2004 | 3301 | 504 | 15.27% |
| RpN4 | May 2016 | 5079105 | 5028279 | 99.00% |
| RpN40 | July 2004 | 101592 | 65727 | 64.70% |
| RpN5 | June 2016 | 4123268 | 1507762 | 36.57% |
| RpN50 | July 2010 | 1864 | 665 | 35.68% |
| RpN51 | July 2007 | 2130607 | 2092445 | 98.21% |
| RpN53 | July 2007 | 1377 | 459 | 33.33% |
| RpN54 | July 2007 | 4018278 | 3988600 | 99.26% |
| RpN55 | July 2007 | 2173309 | 1969894 | 90.64% |
| RpN56 | July 2013 | 3186214 | 2645666 | 83.03% |
| RpN57 | July 2013 | 4055049 | 3568421 | 88.00% |
| RpN58 | July 2013 | 500 | 184 | 36.80% |
| RpN59 | July 2013 | 652144 | 631898 | 96.90% |
| RpN6 | July 2016 | 8208385 | 8131345 | 99.06% |
| RpN60 | July 2013 | 1209 | 729 | 60.30% |
| RpN61 | July 2004 | 2463628 | 2440920 | 99.08% |
| RpN62 | July 2004 | 1138684 | 45692 | 4.01% |
| RpN64 | July 2004 | 3113092 | 2249116 | 72.25% |
| RpN68 | July 2007 | 1900239 | 1882661 | 99.07% |
| RpN69 | July 2007 | 1753805 | 1740003 | 99.21% |
| RpN7 | July 2016 | 7854931 | 6955765 | 88.55% |
| RpN70 | August 2007 | 1591 | 601 | 37.77% |
| RpN71 | July 2010 | 5065311 | 228746 | 4.52% |
| RpN72 | July 2010 | 3362448 | 2508245 | 74.60% |
| RpN73 | July 2010 | 4570586 | 3943462 | 86.28% |
| RpN74 | July 2010 | 4064388 | 2691465 | 66.22% |
| RpN75 | July 2010 | 1300 | 1228 | 94.46% |
| RpN76 | July 2013 | 3733310 | 2027461 | 54.31% |
| RpN77 | July 2013 | 1224125 | 14736 | 1.20% |
| RpN78 | July 2013 | 1206 | 666 | 55.22% |
| RpN8 | July 2016 | 6336591 | 6207972 | 97.97% |
| RpN80 | July 2013 | 3910976 | 62157 | 1.59% |
| RpN9 | July 2016 | 7856312 | 7751657 | 98.67% |
| RpP1 | May 2016 | 5702474 | 5635118 | 98.82% |
| RpP10 | July 2016 | 728 | 3 | 0.41% |
| RpP11 | October 2016 | 6028232 | 5956261 | 98.81% |
| RpP12 | October 2016 | 220 | 1 | 0.45% |
| RpP13 | October 2016 | 4724102 | 4471533 | 94.65% |
| RpP14 | October 2016 | 5222045 | 5155751 | 98.73% |
| RpP15 | October 2016 | 4580228 | 4367378 | 95.35% |
| RpP16 | July 2016 | 7669657 | 7605084 | 99.16% |
| RpP2 | May 2016 | 4120232 | 4081665 | 99.06% |
| RpP22 | October 2013 | 3948582 | 3500291 | 88.65% |
| RpP27 | July 2010 | 462 | 1 | 0.22% |
| RpP28 | July 2010 | 2484910 | 645630 | 25.98% |
| RpP29 | July 2010 | 3349768 | 3097709 | 92.48% |
| RpP3 | May 2016 | 5774855 | 5736933 | 99.34% |
| RpP30 | July 2010 | 12 | 0 | 0.00% |
| RpP31 | July 2010 | 30 | 0 | 0.00% |
| RpP4 | May 2016 | 6570900 | 6217932 | 94.63% |
| RpP40 | October 2013 | 5735515 | 5231599 | 91.21% |
| RpP5 | May 2016 | 3588698 | 3265486 | 90.99% |
| RpP6 | July 2016 | 4951705 | 2230109 | 45.04% |
| RpP7 | July 2016 | 7097326 | 2766864 | 38.98% |
| RpP8 | July 2016 | 5075651 | 4739580 | 93.38% |
| RpSX1 | April 2016 | 36 | 1 | 2.78% |
| RpSX100 | July 2010 | 2681980 | 12496 | 0.47% |
| RpSX101 | July 2010 | 1314275 | 54636 | 4.16% |
| RpSX103 | July 2013 | 1513767 | 45294 | 2.99% |
| RpSX104 | July 2013 | 395897 | 28184 | 7.12% |
| RpSX105 | July 2013 | 3080947 | 48005 | 1.56% |
| RpSX106 | July 2013 | 2581532 | 8291 | 0.32% |
| RpSX11 | October 2016 | 2357899 | 2323869 | 98.56% |
| RpSX13 | October 2016 | 5910183 | 5766040 | 97.56% |
| RpSX24 | October 2013 | 5205780 | 89798 | 1.72% |
| RpSX27 | July 2010 | 2317087 | 1251627 | 54.02% |
| RpSX3 | May 2016 | 5030871 | 4974208 | 98.87% |
| RpSX37 | May 2016 | 6389272 | 5198434 | 81.36% |
| RpSX38 | May 2016 | 7059250 | 7010900 | 99.32% |
| RpSX39 | May 2016 | 3568984 | 3518168 | 98.58% |
| RpSX40 | July 2016 | 6079061 | 4970569 | 81.77% |
| RpSX41 | July 2016 | 7696800 | 7433991 | 96.59% |
| RpSX42 | July 2016 | 9045117 | 8886832 | 98.25% |
| RpSX43 | July 2016 | 6952809 | 3405164 | 48.98% |
| RpSX44 | October 2016 | 6070685 | 5919963 | 97.52% |
| RpSX45 | October 2016 | 6235064 | 5914984 | 94.87% |
| RpSX46 | October 2016 | 5714428 | 5628214 | 98.49% |
| RpSX52 | October 2013 | 7703013 | 7259462 | 94.24% |
| RpSx54 | October 2013 | 6777513 | 6360915 | 93.85% |
| RpSX55 | October 2013 | 7199403 | 6699893 | 93.06% |
| RpSX56 | October 2013 | 5315374 | 3657532 | 68.81% |
| RpSX58 | July 2010 | 14 | 0 | 0.00% |
| RpSX59 | July 2010 | 507165 | 4961 | 0.98% |
| RpSX60 | July 2010 | 2876233 | 20172 | 0.70% |
| RpSX64 | October 2010 | 2513020 | 2023064 | 80.50% |
| RpSX66 | July 2010 | 6692363 | 577943 | 8.64% |
| RpSX68 | July 2004 | 1272256 | 73603 | 5.79% |
| RpSX69 | July 2004 | 2401744 | 1322919 | 55.08% |
| RpSX70 | July 2004 | 3314172 | 281289 | 8.49% |
| RpSX71 | July 2004 | 4365532 | 544744 | 12.48% |
| RpSX72 | July 2013 | 2412080 | 1132019 | 46.93% |
| RpSX74 | July 2013 | 667371 | 27545 | 4.13% |
| RpSX75 | July 2013 | 245196 | 62780 | 25.60% |
| RpSX76 | July 2013 | 739755 | 7057 | 0.95% |
| RpSX77 | July 2007 | 202720 | 16019 | 7.90% |
| RpSX78 | July 2007 | 532 | 16 | 3.01% |
| RpSX79 | July 2007 | 1085301 | 324270 | 29.88% |
| RpSX80 | July 2007 | 764577 | 288423 | 37.72% |
| RpSX81 | July 2007 | 770022 | 48394 | 6.28% |
| RpSX82 | July 2010 | 1573104 | 111450 | 7.08% |
| RpSX83 | July 2010 | 341770 | 35717 | 10.45% |
| RpSX84 | July 2010 | 1970780 | 33920 | 1.72% |
| RpSX85 | July 2010 | 1316713 | 600969 | 45.64% |
| RpSX86 | July 2010 | 4019654 | 3163892 | 78.71% |
| RpSX87 | July 2004 | 6052227 | 4697286 | 77.61% |
| RpSX88 | July 2004 | 6848215 | 4988476 | 72.84% |
| RpSX89 | July 2004 | 3297635 | 59740 | 1.81% |
| RpSX9 | July 2016 | 8279828 | 7767546 | 93.81% |
| RpSX90 | July 2004 | 2916365 | 1644707 | 56.40% |
| RpSX91 | July 2004 | 3215721 | 1395703 | 43.40% |
| RpSX92 | July 2007 | 11973 | 8607 | 71.89% |
| RpSX93 | July 2007 | 889 | 787 | 88.53% |
| RpSX94 | July 2007 | 1879146 | 856579 | 45.58% |
| RpSX95 | July 2007 | 2014436 | 62310 | 3.09% |
| RpSX96 | July 2007 | 1121611 | 711733 | 63.46% |
| RpSX97 | July 2010 | 2404596 | 7163 | 0.30% |
| RpSX98 | July 2010 | 2810450 | 63968 | 2.28% |
| RpSX99 | July 2010 | 1982435 | 11898 | 0.60% |
| RpW1 | April 2016 | 6741040 | 6679197 | 99.08% |
| RpW10 | July 2016 | 6681470 | 6369210 | 95.33% |
| RpW11 | October 2016 | 5196967 | 5147924 | 99.06% |
| RpW12 | October 2016 | 5789812 | 5747978 | 99.28% |
| RpW13 | October 2016 | 6485111 | 6436316 | 99.25% |
| RpW14 | October 2016 | 6306791 | 6264912 | 99.34% |
| RpW15 | October 2016 | 5511667 | 5463819 | 99.13% |
| RpW2 | May 2016 | 5344539 | 4502008 | 84.24% |
| RpW3 | May 2016 | 2126278 | 1156035 | 54.37% |
| RpW4 | May 2016 | 6256548 | 6210680 | 99.27% |
| RpW5 | May 2016 | 5133083 | 5090163 | 99.16% |
| RpW6 | July 2016 | 7284018 | 6302234 | 86.52% |
| RpW7 | July 2016 | 7782377 | 6500144 | 83.52% |
| RpW8 | July 2016 | 7759623 | 4786033 | 61.68% |
| RpW9 | July 2016 | 6941326 | 6621067 | 95.39% |
| RpWr12 | July 2016 | 2025720 | 2008870 | 99.17% |
| RpWr13 | July 2016 | 1896207 | 1881431 | 99.22% |
| RpWr14 | July 2016 | 2433675 | 2414126 | 99.20% |
| RpWr15 | July 2016 | 1911181 | 1896378 | 99.23% |
| RpWr16 | August 2016 | 1811875 | 1797407 | 99.20% |
| RpWr19 | August 2016 | 1870903 | 1855521 | 99.18% |
| RpWr22 | July 2016 | 1513893 | 1496810 | 98.87% |
| RpWr23 | July 2016 | 2055206 | 2035664 | 99.05% |
| RpWr3 | July 2016 | 2220158 | 2117264 | 95.37% |
| RpWr9 | July 2016 | 1943112 | 1845462 | 94.97% |
| RpY11 | July 2016 | 1792378 | 1778307 | 99.21% |
| RpY14 | August 2016 | 2216720 | 2103338 | 94.89% |
| RpY16 | August 2016 | 1833230 | 1816076 | 99.06% |
| RpY21 | August 2016 | 5057783 | 4855700 | 96.00% |
| RpY22 | August 2016 | 1945851 | 1834913 | 94.30% |
| RpY26 | August 2016 | 2115759 | 2096472 | 99.09% |
| RpY27 | August 2016 | 5958699 | 5912133 | 99.22% |
| RpY6 | July 2016 | 3173082 | 3140742 | 98.98% |
| RpY8 | July 2016 | 2026918 | 2007246 | 99.03% |
| RpY9 | July 2016 | 1943929 | 1922756 | 98.91% |
| **Average** |  | 3708552 | 2985915 | 65% |

**Table S5.** Pairwise genetic differentiation (F_ST_) between samples from the North (A) and South (B) locations collected at different seasons in the year. N – Newcastle, P – Preston, Sx – Starcross, W – Wye. Column Ng shows the number of gene copies per sample group (number individuals x 2). Significant values are shown in italics.

**A)**

|  | **Ng** | **N spring** | **N summer** | **N autumn** | **P spring** | **P summer** | **P autumn** |
| --- | --- | --- | --- | --- | --- | --- | --- |
| **N spring** | 8 | - |  |  |  |  |  |
| **N summer** | 30 | *0.057* | - |  |  |  |  |
| **N autumn** | 16 | *0.067* | *0.039* | - |  |  |  |
| **P spring** | 10 | 0.030 | 0.028 | *0.051* | - |  |  |
| **P summer** | 6 | 0.052 | 0.011 | *0.048* | 0.025 | - |  |
| **P autumn** | 8 | *0.073* | *0.061* | *0.042* | *0.061* | *0.063* | - |

**B)**

|  | **Ng** | **W spring** | **W summer** | **W autumn** | **Sx spring** | **Sx summer** | **Sx autumn** |
| --- | --- | --- | --- | --- | --- | --- | --- |
| **W spring** | 10 | - |  |  |  |  |  |
| **W summer** | 10 | -0.038 | - |  |  |  |  |
| **W autumn** | 10 | *0.164* | *0.123* | - |  |  |  |
| **Sx spring** | 8 | 0.032 | 0.003 | *0.235* | - |  |  |
| **Sx summer** | 6 | -0.023 | -0.050 | *0.082* | *0.079* | - |  |
| **Sx autumn** | 10 | 0.005 | -0.013 | *0.102* | 0.040 | -0.020 | - |
