# Supplementary material for: Population genetic structure and predominance of cyclical parthenogenesis in the bird cherry‐oat aphid Rhopalosiphum padi in England
Source: Evol Appl. 2020 Feb 3;13(5):1009–25. doi: 10.1111/eva.12917 (PMC7232763; doi:10.1111/eva.12917)
Supplement: Supplementary file 3 [file EVA-13-1009-s003.docx]

*DNA yield comparison across sample years*

Of the 316 aphids used in the study, 34 samples yielded less than 0.01 ng (Supplementary Table S1). Of these, 19 were samples collected in 2013, 14 in 2010 and 1 aphid individual was from 2016; in addition, all these samples were extracted using Qiagen’s Blood & Tissue kit. Aphids from 2016 yielded on average the highest amount of DNA (382.04 ng ± 278.15 standard deviation), followed by samples from 2007 (311.73 ng ± 410.28), 2004 (203.32 ng ± 145.33), 2010 (80.21 ng ± 124.17) and 2013 (70.54 ng ± 168.64). However, the variation in yield from samples within each year is high and they range from 23.82 ng to 426 ng (2004), 18.6 – 1332 ng (2007), 9.09 - 660 ng (2010), 12.1 – 996 ng (2013), and 16.56 – 1400 ng (2016) (Supplementary Table S1). The amount of DNA obtained from aphids from 2010 and 2013 was significantly smaller than that obtained from 2004, 2007 and 2016 samples (Wilcoxon rank sum test *P* < 0.01, after Bonferroni correction), the average DNA obtained from 2016 samples was significantly higher than that from samples collected in other years (*P* < 0.005 after Bonferroni correction) except 2007 (Wilcoxon rank sum test *P* = 0.2431 after Bonferroni correction), and there is no significant difference between the DNA obtained from samples from 2007 and 2004 (Wilcoxon rank sum test *P* = 1 after Bonferroni correction). In addition, the two different DNA extraction kits used in samples from 2010, 2013 and 2016 provided different amounts of DNA on average, with Qiagen’s DNA Micro kit resulting in higher amounts (Table 1, Figure S2), and significantly different in the samples from 2010 and 2016 (Wilcoxon rank sum test 2010: *P* = 0.024; 2013: *P* = 0.81; 2016: *P* = 7.8 x 10^-11^, with Bonferroni correction).
